# Prevalence and associated factors of low vision and blindness among patients attending St. Paul’s Hospital Millennium Medical College, Addis Ababa, Ethiopia

**DOI:** 10.1186/s12886-018-0899-7

**Published:** 2018-09-03

**Authors:** Fashe Markos Cherinet, Sophia Yoseph Tekalign, Dereje Hayilu Anbesse, Zewdu Yenegeta Bizuneh

**Affiliations:** Department of Ophthalmology, St. Paul’s Hospital Millennium Medical College, Addis Ababa, Ethiopia

**Keywords:** Low vision and blindness, St. Paul’s hospital millennium medical college, Ethiopia

## Abstract

**Background:**

Low vision and blindness are major public health problems. A vast burden of worlds visually impaired live in low-income settings especially in sub Saharan Africa. In such settings the blindness is associated with considerable disability and excess mortality, resulting in huge economic and social consequence. The main purpose of this study was to determine the prevalence and associated factors of low vision and blindness among patients at St. Paul’s hospital millenium medical college.

**Methods:**

Institution based cross sectional design study was carried out from January to April, 2017 with sample size of 904. Systematic random sampling was used to recruit the study subjects. Retrospective medical chart review was done; data was entered into and analyzed by SPSS 23. Descriptive statistics such as frequency cross tabulation and chi-square test was carried out to translate data into information. *P*-value less than 0.05 was considered as statistically significant.

**Results:**

A total of 881 subjects with a response rate of 97.4% selected. The mean age of the study subjects was 44.53(SD: ± 21.85) with a range of 1–100 years. The prevalence of low vision and blindness was 91 (10.3% (95% CI: 8.2, 12.3)), and 64 (7.3 95%CI: 5.7, 9.0)) respectively. Age (*p*-value < 0.001), cataract (*p*-value = 0.002), glaucoma (*p*-value = 0.002) and age related macular degeneration (*p*-value < 0.001) were significantly associated with low vision and blindness.

**Conclusion:**

Low vision and blindness found in this study was high. Age, cataract, glaucoma and age related macular degeneration were significantly associated with low vision and blindness. This amount of magnitude will be reduced if prevention, early diagnosis and management will be targeted towards avoidable causes of visual impairment.

## Background

In the world, the number of visually impaired is estimated to be 285 million, of whom 39 million were blind. About 82% of peoples greater than 50 years and above were blind and this indicates that the burden of visual impairment and blindness is higher on the older age groups [1]. Visual impairment includes low vision and blindness. It was defined based on visual acuity and visual field [2].

A vast burden of worlds visually impaired live in low-income settings especially in sub Saharan Africa (SSA) [3]. Visual impairment can cause disabilities by significantly interfering with one’s ability to function independently. These disabilities limit personal or socioeconomic independence and a visual handicap exists [4]. The good news is that more than two third of this visual impairment and blindness can be avoidable either by prevention and treatment [5].

According to ‘vision 2020 the right to sight’ plan, in 2020 preventable and avoidable causes of visual impairment and blindness will be reduced significantly to less than 0.5% in all countries or less than 1% in any community worldwide [6–9].

Ethiopia is believed to have one of the world’s highest rates of blindness (1.6%) and low vision (3.7%), of which more than 80% is either treatable or preventable [2, 10–12]. Low vision and blindness are believed to be common among patients in St. Paul’s Hospital Millennium Medical College, though no survey was conducted in the setting. This survey was conducted with the main objective of assessing the prevalence and associated factors of low vision and blindness among patients in St. Paul’s Hospital Millennium medical College (SPHMMC), Addis Ababa; Ethiopia. Thus, this evidence will help us to establish low vision and blindness rehabilitation clinic in the hospital.

## Methods

### Study design, setting and sampling

An institution based- cross sectional design study was conducted from January to April 2017 at SPHMMC, Tertiary Eye Care and Training Center. The center is well known college with ophthalmic medical service and ophthalmology residency training in the capital city of the country, Addis Ababa. It has been serving more than 16,000 patients with more outpatients and in some cases inpatient services per year. There are sub specialty clinics like retina, glaucoma, pediatric, oculoplasty and optometry clinics within the center. All patients come to the center have been undergoing complete eye examination including diagnostics tests. Medical, optical, laser and surgical service is the routine treatment modalities for the patients at the center. There are also one extra public and two private tertiary eye care centers in the study area. The other 10 public and 15 private secondary and primary eye centers were more focused on treating adnexal eye diseases and major surgeries like cataract and Trabeculectomy, minor surgeries and refractive services.

There was no evidence within the same study setting in which we need to consider in estimating the minimum sample size. Therefore it was considered that 50% proportion of population with visual impairment, 3% error of margin, 95% confidence interval and 10% non- respond rates were used to calculate the sample. Hence a total of 904 sample size was retrieved using single population proportion formula. The study was conducted among persons of all age groups. Study subjects were recruited from registration logbook of the clinic taking every 10th patient, the first being the 7^th^medical record number registered with omission of incomplete records.

Socio-demographic characteristics such as age, sex and address and clinical characteristics like Snellen visual acuity, IOP measured by non-contact air-puff tonometer, slit lamp biomicroscope and Fundus examination results including lists of all ophthalmic diagnosis written on the medical record were retrieved as their respective order. Visual acuity and IOP were measured by ophthalmic nurses while eye examinations were done by senior ophthalmologists. Visual acuity was measured by using retro-illuminated Snellen charts at 6 m and only distance one was considered; and for children less 4 years of age HOTV cards was used. The presenting visual acuity was taken in all cases and for children less than 4 years age appropriate visual acuity equivalent recorded for analysis. Data were recorded on a customized form, and the cause of visual impairment determined from patient cards with primary diagnosis was taken for all participants with a presenting VA of less than 6/18 for each eye separately.

### Operational definition

The WHO categories of visual impairment were used to define vision status for study participants. Blindness was defined as a presenting VA of less than 3/60 in the better eye. Low vision was defined as presenting VA of at least 3/60 but less than 6/18 in the better eye. Monocular visual impairment, which is not a WHO definition, was derived to represent participants who had normal or near-normal vision in the better eye (VA of at least 6/18) and visual impairment in the other eye (VA from 6/18 up to 3/60 for low vision and less than 3/60 for blindness) [13].

### Data quality assurance and ethical clearance

After the survey, the investigators reviewed the forms and determined the principal disorder responsible for blindness or low vision for the participant, taking into account the main cause for each individual eye. In the instance when different causes had been identified for each eye separately in a given individual, the principal disorder was chosen to be the one that was most readily curable or, if not curable, most easily preventable. The study was conducted in accordance with declaration of Helsinki and approved by institutional review board of SPHMMC according to Ethiopian national research ethics review guideline. The privacy and confidentially of all subject was secured. The department’s usual data quality control is just keeping patients’ clinical profile on log book at each clinic. The data for this study was retrieved from those log-books. Institutional review board of SPHMMC stated that obtaining informed consent for participation is not applicable the study based on the secondary data.

### Statistical analysis

The collected data was entered twice, carefully cleaned and analyzed using SPSS version 23 (www.ibm.com/products/spss-statistics). Descriptive statistics such as frequency distribution and central tendency measures were used to summarize the descriptive part of the study. Pearson X^2^ was used to determine the factors associated with low vision and blindness [14]. *P*-value less than 0.05 were considered as statistically significant.

## Results

A total of 881 study subjects with a response rate of 97.4% were recruited in the study. The study subjects have a mean age of 44.53(SD: ± 21.85), median of 48 with a range of 1–100 years. Among those subjects a quarter 225(25.5%) of them were in the age group of 1–26 years and about half 432(49.0%) of them were male in sex. More than three fourth 684(77.6%) of total subjects address were in urban Table 1.

**Table 1:** Socio-demographic characteristics of study subjects among patients attending St. Paul’s Hospital Millennium Medical College, 2017 (*n* = 881)

Variables	Frequency	Percent
Age		
1–26	225	25.5
27–48	227	25.8
49–62	221	25.1
63–100	208	23.6
Sex		
Male	432	49.0
Female	449	51.0
Address		
Urban	684	77.6
Rural	197	22.4

Among a total study subjects, greater than four fifth 726 (82.4%) of patients have visual acuity in the range of 6/6–6/18 and more than three fourth 470 (77.9%) of patients have intraocular pressure within the range of 9–21 mmHg Table 2.

**Table 2:** Clinical Characteristics of study subjects among patients attending St. Paul’s Hospital Millennium Medical College, 2017

Variables	Frequency	Percent
Visual acuity(*n* = 881)		
6/6–6/18	726	82.4
6/24–3/60	91	10.3
< 3/60	64	7.3
Intraocular pressure in mmHg(*n* = 603)		
< 8	3	0.5
9–21	470	77.9
> 21	130	21.6

Of all study subjects about one fifth of them were presented with cataract 181 (20.5%) followed by glaucoma 158 (17.9%) and refractive error 118 (13.4%) Table 3.

**Table 3:** Disease pattern of study subjects among patients attending St. Paul’s Hospital Millennium Medical College, 2017 (*n* = 881)

Diseases	Frequency (%)	Presenting VA < 6/18–3/60 (%)	Presenting VA < 3/60 (%)
Cataract	181(20.5)	31(34.1)	15(23.4)
Glaucoma	158(17.9)	20(22.0)	21(23.4)
Refractive error	118(13.4)	14(15.4)	3(4.7)
Pseudophakia	111(12.6)	20(22.0)	6(9.4)
Trauma	72(8.2)	1(1.1)	8(12.5)
ARMD	29(3.3)	11(12.1)	12(18.8)
Strabismus	15(1.7)	2(2.2)	0(0.0)
DR	10(1.1)	1(1.1)	2(3.1)
HR	6(0.7)	0(0.0)	0(0.0)
Uveitis	11(1.2)	1(1.1)	2(3.1)
TCO	6(0.7)	1(1.1)	1(1.6)
NTCO	14(1.6)	2(2.2)	1(1.6)
Others	317(36.0)	1(1.1)	4(6.2)

*ARMD* age related macular degeneration, *DR* diabetic retinopathy, *HR* hypertensive retinopathy, *TCO* trachomatous corneal opacity, *NTCO* non-trachomatous corneal opacity

A total of 155 (17.6% (95% CI: 15.2, 20.1)) subjects were visually impaired depending on the presenting visual acuity. Among the study subjects, 91 (10.3% (95% CI: 8.2, 12.3)) had low vision and 64 (7.3 95%CI: 5.7, 9.0)) blindness. One hundred sixteen (13.2% (95% CI 10.9, 15.6)) and 170(19.3% (95% CI 17.0, 22.1)) had monocular low vision and blindness respectively.

Low vision and blindness were 42(20.2%) and 34(16.3%) in age group between 63 and 100 years old respectively. Among the subjects presented with refractive error, 14 (11.9%) and 3(2.5%) had low vision and blindness whereas 1(1.4%) and 8(11.1%) traumatic patients had low vision and blindness respectively. The factors such as age (*p*-value < 0.001), cataract (*p*-value = 0.002), glaucoma (*p*-value = 0.002) and age related macular degeneration (*p*-value < 0.001) were significantly associated with low vision and blindness Table 4.

**Table 4:** The factors associated with low vision and blindness among patients attending St. Paul’s Hospital Millennium Medical College, 2017 (*n* = 881)

Variables	Normal	Low vision	Blindness	X^2^	*p*-value
Age				91.76	0.000
1–26	221(93.8%)	8(3.6%)	6(2.7%)		
27–48	210(92.5%)	10(4.4%)	7(3.1%)		
49–62	173(78.3%)	31(14.0%)	17(7.7%)		
63–100	132(63.5%)	42(20.2%)	34(16.3%)		
Sex				3.99	0.14
Male	348(80.6%)	45(10.4%)	39(9.0%)		
Female	378(84.2%)	46(10.2%)	25(5.6%)		
Address				1.38	0.50
Urban	566(82.7%)	72(10.5%)	46(6.7%)		
Rural	160(81.2%)	19(9.6%)	18(9.1%)		
Cataract				12.21	0.002
No	591(84.4%)	60(8.6%)	49(7.0%)		
Yes	135(74.6%)	31(17.1%)	15(8.3%)		
Glaucoma				12.27	0.002
No	609(84.2%)	71(9.8%)	43(5.9%)		
Yes	117(74.1%)	20(12.7%)	21(13.3%)		
Refractive error				4.66	0.1
No	625(81.9%)	77(10.1%)	61(8.0%)		
Yes	101(85.6%)	14(11.9%)	3(2.5%)		
Pseudophakia				8.40	0.02
No	641(83.2%)	71(9.2%)	58(7.5%)		
Yes	85(76.6%)	20(18.0%)	6(5.4%)		
Trauma				7.91	0.02
No	663(82.0%)	90(11.1%)	56(6.9%)		
Yes	63(87.5%)	1(1.4%)	8(11.1%)		
ARMD				84.02	0.000
No	720(84.5%)	80(9.4%)	52(6.1%)		
Yes	6(20.7%)	11(37.9%)	12(41.4%)		
Strabismus				1.28	0.53
No	713(82.3%)	89(10.3%)	64(7.4%)		
Yes	13(86.7%)	2(18.3%)	0		
DR				2.45	0.29
No	719(82.5%)	90(10.3%)	62(7.1%)		
Yes	7(70.0%)	1(10.0%)	2(20.0%)		
HR				1.29	0.53
No	720(82.3%)	91(10.4%)	64(7.3%)		
Yes	6(100%)	0	0		
Uveitis				0.97	0.38
No	718(82.5%)	90(10.3%)	62(7.1%)		
Yes	8(72.7%)	1(9.1%)	2(18.2%)		
TCO				1.15	0.56
No	722(82.5%)	90(10.3%)	63(7.2%)		
Yes	4(66.7%)	1(16.7%)	1(16.7%)		
NTCO				0.24	0.89
No	715(82.5%)	89(10.3%)	63(7.3%)		
Yes	11(78.6%)	2(14.3%)	1(7.1%)		
Others				88.05	0.000
No	414(73.4%)	90(16.0%)	60.3(10.6%)		
Yes	312(98.0%)	1(0.3%)	4(1.3%)		

*ARMD* age related macular degeneration, *DR* diabetic retinopathy, *HR* hypertensive retinopathy, *TCO* trachomatous corneal opacity, *NTCO* non-trachomatous corneal opacity

## Discussion

Visual impairment, which includes low vision and blindness, remain a public health problem that has impact on socioeconomic values and quality of life of the community. This study was targeted to determine the magnitude and factors of visual impairment under the category of low vision and blindness so that stakeholders will have evidence to plan and implement prevention and management strategies in the hospital and surrounding communities.

In the present study the prevalence of visual impairment was 155 (17.6% (95% CI: 15.2, 20.1)). This finding is higher than other studies conducted within the communities [12, 15, 16]. This indicated that hospital burden of visual impairment is the reflective of community problems. It might also revealed that the patients comes to hospital is usually after their visual function is severely affected which is the common problems of low income population. It is lower than study conducted in South Africa (28.0%) and Ghana (28.2%) [17, 18]. The discrepancy observed here might be due to sampling technique in which they used non-probability sampling. The population difference and eye care service difference at the two centers might also contribute for the different proportion of visual impairment.

Among the study subjects, 91 (10.3% (95% CI: 8.2, 12.3)) had low vision and 64 (7.3 95%CI: 5.7, 9.0)) blindness. This finding indicated that the low vision and blindness is a major public health problem. In comparison to national survey result of low vision (3.7%) and blindness (1.6%) conducted 2005 in Ethiopia this finding is higher as it is hospital based unlike national survey [10]. Though 3 years left to the Vision to 2020 and ‘the right to sight’ goal, in this study area the burden of the low vision and blindness still high and need strengthening the prevention of avoidable causes of low vision and blindness. This result is consistent with similar study conducted in Gondar Ethiopia (15.3%) [19].

The prevalence of blindness in the study is lower than studies conducted in South Africa (10.9%) and Kenya (39.4%), higher than studies conducted in Cameroon (1.71%) but in line with study reported form Mali (5.8%) and Jordan (13.7%) [17, 20–23]. The low vision is consistent with study report from Nigeria (9.2%) and Cambodia (12%) [24, 25], but lower than South African study (16.3%) [17]. This reflects that there are a lot of factors that can contribute for geographical variations of low vision and blindness such as socio-economic difference, climatic change, gene and ethnical difference, health care service system, number of eye care givers and supportive organizations and not all of those could be investigated in the present study.

The amount of monocular low vision and blindness in this study was (13.2% (95% CI 10.9, 15.6)) and 170(19.3% (95% CI 17.0, 22.1)) respectively. As the worst visual acuity was considered to define monocular low vision and blindness, it is expected to be higher than bilateral one. This result is higher than study done in Thailand, which reported 3.0% low vision and 4.4% blindness [26]. The discrepancy observed here might be due to different socioeconomic values and eye care seeking behaviors.

The factors associated with low vision and blindness in the current study were age (<*p* < 0.001), cataract (*p* = 0.002), glaucoma (*p* = 0.002) and age related macular degeneration (*p* < 0.001). Age was reported from different clinical and community based studies as the main risk factors for visual impairment, low vision and blindness [27, 28]. However gender was not associated with low vision or blindness like study conducted in Australia [29]. The eye conditions such as cataract, glaucoma, and age related macular degeneration were also reported as the main etiology or causes of low vision and blindness [30]. Factors such as level of education, inability to afford service cost, fear of the outcome of the surgery especially for cataract and glaucoma and cultural beliefs are some of the reason why people remain low in vision and blindness. Those diseases are among either preventable or avoidable disease if they are diagnosed and treated early.

In addition to that uncorrected refractive error, ocular trauma and Pseudophakia were also among the major causes of low vision and blindness. These finding were also reported by different studies in Ethiopia from national survey and Gurage zone [11].

There was some limitation with this study. Most importantly different associated factors were not well explored, as it was hospital based and used secondary data from patients’ medical record.

## Conclusion

Low vision and blindness found in this study was high. Age, cataract, glaucoma and age related macular degeneration were significantly associated with low vision and blindness. This amount of magnitude will be reduced if prevention, early diagnosis and management will be targeted towards avoidable causes of visual impairment.

## Abbreviations

SPHMMC: St. Paul’s Hospital Millennium Medical College
VA: Visual acuity
WHO: World Health Organization
